# Static and Dynamic Balance Indices among Kindergarten Children: A Short-Term Intervention Program during COVID-19 Lockdowns

**DOI:** 10.3390/children9070939

**Published:** 2022-06-22

**Authors:** Einat Yanovich, Salit Bar-Shalom

**Affiliations:** The Academic College at Wingate, Netanya 4290200, Israel; salinka27@gmail.com

**Keywords:** COVID-19, lockdown, motor learning, motor skills, balance

## Abstract

The COVID-19 pandemic outbreak had a negative impact on kindergarten activities. These young children, who had been compelled to stay home during lockdowns, suffered a lack of movement and loss of mobility, resulting in deteriorated physical motor skills. Lack of sufficient motor experience in early childhood can impair children’s motor and cognitive development. Balance skills are fundamental to all other motor abilities, from the most basic movements to the most complex motor skills. The purpose of this study was to implement a short-term physical activity program, which may have a direct effect on children’s fundamental balance ability. Ninety-six kindergarten children (45 boys and 51 girls), aged 4–6 years, participated in the study. Data were analyzed using three-way ANOVA and interaction analyses. The results suggest that short, focused, and dedicated balance training programs have a beneficial influence on the static balance of preschoolers and can mitigate some of the negative physical outcomes of lockdowns. In conclusion, this study indicates that a short-term physical training program had a positive effect on the motor abilities of preschoolers after COVID-19-related lockdowns. More research is needed in order to fully understand the complete impact of the worldwide health crisis and the best ways in which to address it.

## 1. Introduction

The term “balance” refers to a type of motor movement coordination in which the visual and kinesthetic components of the body muscles work together with the balance sensors that are located in the middle ear in order to maintain the body’s stability without unnecessary movements or falling [1]. The ability of body balance depends on internal systems, such as the vestibular system (equilibrium sensors), the proprioceptive system (responsible for motion sensors), and the visual system. Balance also depends on certain external factors, such as the support base, the center of gravity, and the body’s structure and weight [2].

In the field of movement, the ability of two-legged creatures to stabilize themselves is especially important and requires being perpendicular from the center of gravity and its supporting area, i.e., standing on their legs and the area between their legs. Moving from standing on one leg to standing on the other requires advancement along with a narrow basis while maintaining stability and activating the balance system as part of the posture [3]. In addition, basic movements such as running, changing direction, stopping, and advancing on elevated surfaces require the maintaining of the person’s stability and necessitate training for developing the balance system [4].

Balance skills serve as the basis of all other motor abilities, from basic movements to the most complex motor skills [5]. The muscular-nervous system even constitutes a connection point for certain cognitive processes, such as attention, concentration, and mental imagery [6]. A correlation was found between body balance and mental states, such as the sense of self-efficacy, depression, and anxiety [7].

The ability to maintain balance is subject to change during a person’s lifespan, such as changes in body structure, experience in the given activity, and complex challenges [8].

### 1.1. Types of Balance

There are two types of balance: static and dynamic. Static balance takes place when the center of gravity is maintained vertically above the base, without changing the base lengthwise. Static balance is maintained as long as the pressure on the organs that are carrying the body weight is consistently located at the center of the body mass [9]. Humans can choose the base of support, its size, and how the body is structured on it [10]. Maintaining balance is more challenging when the base of support is smaller, has a greater slope, and is less stable, and if the individual has a higher center of gravity [4]. Examples of static balance include standing on one foot and standing in an elevated position on one’s feet.

The term dynamic balance relates to the ability to maintain the center of gravity above the base during movement, with the body exiting the center of gravity. In dynamic balance, the main process is the coordination between holding the torso above the center of gravity and various advancement movements, which allows for stability and reflectivity in reaction to changes that occur during the movement. To successfully maintain dynamic balance, one must be prepared with responses to expected alterations [11]. Dynamic balance is part of any skill of progress and is manifested in the moment of transition from base to base when there is a detachment of a part of the body moving from the ground. Examples of detachment are walking, running, jumping, and landing.

### 1.2. The Importance of Balance in Preschool Children

Significant maturity of the nervous system cannot be achieved in preschool children, even when they are going through normal and substantial motor development processes if routine experiences of certain key movements are lacking (as in the case of lockdown). The muscular nervous system, which enables movement coordination, is acquired and developed from the embryonic stage onwards [12]. Early childhood presents a window of opportunity for motor development, yet insufficient motor experience can impair motor development among children and even later in life. The system’s requirements for creating efficient movements are complex, yet the prevailing assumption is that such requirements serve as the main factors in the development of the balance system during childhood and in the importance of abundant movement experience [5].

Balance ability is the basic condition for the performance of motor skills, and it depends on internal and external systems. Motor experience in ages 3–7 is a necessary condition and a key factor for developing synchronization between various systems. Nevertheless, it is difficult to indicate the exact age at which the ability to balance ability reaches maturity or peak functionality since the developmental processes of balance are not consistent. Balance sensors, for example, are the last to evolve due to the complexity of this system and its dependence on other sensory systems. As such, toddlers and young children mainly use motion and vision sensors to maintain balance, only adding balance sensors later on in their development [13].

Throughout this developmental process, there are certain milestones that should be pointed out. During the first weeks of life, babies practice using their balance sensors by trying to lift the upper part of their torso, neck, and head while lying on their stomachs. From the second half of infancy (i.e., from six months and onwards) and towards the end of this period, children are able to perform activities such as crawling, standing, and slow walking. However, these activities cannot be performed without initial coordination between vision, motion, and balance sensors. From the moment infants begin walking, this system receives a significant developmental boost since this skill requires constant adaptation of the body to movement alterations in space and to different situations, resulting in the development of balance sensors [14].

In independent walking, children demonstrate control of their torso volatility for the first time, as previous skills required them to concentrate on controlling their body balance above the base [15]. The important development of the child’s balance ability at the kindergarten age advances at a rapid pace following ample practice. Educational frameworks for ages 3–7, therefore, should monitor the development of this ability among the children in order to ensure continuous improvement [16].

### 1.3. The Effect of the COVID-19 Pandemic

Following the outbreak of the COVID-19 pandemic in Israel in March 2020, educational institutions across Israel closed their doors—including kindergartens, rendering more than half a million children aged 3–6 homebound and in social isolation. As kindergartens provide a supportive physical and cognitive environment for the acquisition and development of cognitive, emotional, social, and physical skills among young children, short-term effects of the lockdown on educational, personal, and social aspects rapidly emerged [17,18,19]. The long-term effects, however, are yet to be fully observed and explored.

Quarantine phases have a widespread effect on the acquisition and establishment of knowledge and skills. Significant developmental milestones and processes that take place in kindergarten could have been damaged by the long lockdown, with the possible forming of gaps and inequality processes. There is already evidence as to longer screen time and less physical activity among children [19], as well as poorer balance [20]. Gaps in social, sensory-motor, language, life, and motor skills have also been documented [17]. Long periods of quarantine may also alter the way children play and affect their social behavior [21]. There is also concern that if not adequately addressed, these gaps in development may affect children’s acquisition of developmental indicators in the future as well.

Static balance tends to decrease between the end of kindergarten and the end of the first year at school (ages 5–7) [22]. Deterioration may worsen in times of emergency lockdown. Without noteworthy interventions, such gaps may continue to expand, in addition to primary deficits that are already showing indications. The education system in Israel faced three remote-learning periods during the crisis, with 4–8 weeks of grace between each of these periods. The first period lasted 8 weeks, the second 6 weeks, and the third remote-learning period lasted 5 weeks. During these periods, all parks, playgrounds, and recreation areas were closed. As such, educators found themselves attempting to achieve as much as possible during the infrequent periods when the children physically attended kindergartens and schools.

In light of this background, the aim of this study was to implement a short, targeted program that could have a direct effect on children’s fundamental balance ability. The study hypothesizes that this short-term program will make a beneficial contribution to children’s static and dynamic balance.

## 2. Materials and Methods

### 2.1. Participants

The study included 96 kindergarten children (45 boys and 51 girls), aged 4–6 years, from four different kindergartens in a central district in Israel. The four kindergartens were all situated on one site and belonged to the same cluster, which was chosen through convenience sampling. This kindergarten cluster (“Eshkol Ganim” in Hebrew) is characterized by a medium-high socioeconomic status and serves the majority of children in this geographical area. All four kindergartens are structured similarly. Each building housing a kindergarten consists of two large rooms; one has a table and chairs for educational activities such as painting and art, and the other is used for gatherings and for free play. The outside yard of each kindergarten is equipped with a sandbox, a ladder and slide, and a swing.

The four kindergartens were cluster-randomized, whereby two kindergartens were randomly assigned to the control group (*n* = 46; age: 4.8 ± 1.63), and the remaining two were assigned to the study group (*n* = 50; age: 4.63 ± 1.12). This allocation was mandatory since the study was conducted by the physical education teacher as part of the regular curriculum in which each kindergarten receives only one hour per week of physical education, and since the COVID-19 health regulations disallowed the mixing of classes. Anthropometric data (height and weight) were not collected since the study was conducted as part of the kindergarten’s routines and had to follow the formal restrictions. The Israeli Ministry of Education prohibits taking any body measurements in all the institutions under its inspection. The inclusion criterion was participating in all study sessions. Children who missed one or more sessions were not included in the sample, although they continued to participate in the classes. The kindergarten teachers had no previous knowledge of the study’s goals.

### 2.2. Procedure

After receiving the Institutional review board agreement, parents’ consent forms, and permission from the district’s kindergartens supervisor, the study began. Data collection in all kindergartens was conducted by the same experimenter during the day’s activities, while the daily routine was kept fixed.

In the pre-intervention phase (T0—one session), the experimenter met with the kindergarten teachers and children and evaluated the static and dynamic balance of each child via a battery of motor tests described below. The tests were conducted in an isolated room in the kindergarten, which had been specifically pre-assigned for this process. The resting time between each motor test was two minutes (passive break) to allow the children to relax. Two children were tested intermittently; one performed the motor tests, while the other rested. The order of the motor tests was identical for all children, and the kindergarten staff was not involved in the data collection process. The encounters lasted 45 min each and were conducted throughout the regular kindergarten day, from 8 am to 1:30 p.m.

The intervention phase (T1—three sessions) entailed balance training, as presented in Appendix A. Both the experimental group and the control group underwent three 45-minute sessions, with seven days between each session. This study design was selected as a weekly session of 45-minute physical education is the standard curriculum. During these interventions, both dynamic and static balance training was taught to the experimental group, while general physical activities (e.g., walking at a changing pace, dancing, moving, and stopping) with no accentuation of balance were carried out by the control group. The balance training consisted of 10 minutes of warming up, 30 min of balance routines (3–5 repetitions of each routine), and 5 minutes of relaxing and summarizing the session. A physical education teacher for preschoolers conducted the interventions in both study groups and maintained the same methodology.

Post-intervention assessments (T2—one session) were carried out during the fifth and final session. These assessments were identical to those carried out during the first session in the pre-intervention phase. Following the completion of the study, and after all measurements were taken, the control group then received the balance training, and the experimental group received the regular physical activity sessions as part of their physical education classes.

### 2.3. Research Tools

To examine the impact of the intervention, the pre- and post-intervention tests included two motor tests: the one-leg stance (stork balance stand test) [23] and the one-leg hop test [24]. The one-leg stance evaluates the individual’s ability to stand on one leg for as long as possible, as measured in seconds. The test’s reliability depends on how strictly the test is conducted combined with the individual’s level of motivation to perform. Test validity, calculated based on published tables regarding fitness level, indicated high correlations [25]. In this study, the children were instructed to stand on one leg for as long as possible, with parallel thighs and with the opposite knee elevated to 90°. The best measurement out of three was documented for each leg. The one-leg hop test evaluates dynamic balance and has been shown to have a strong intrarater reliability, with an intraclass correlations coefficient of 0.85 for both legs. In this study, the children were asked to hop on one leg as many times as they could without stopping between hops and then to repeat the exercise on the other leg. The best measurement out of three was documented for each leg.

### 2.4. Statistical Analysis

Data analysis was performed using SPSS^®^ version 26.0 for Windows (IBM Corp, Armonk, NY, USA). Comparisons between the groups at the different time points were conducted using a three-way analysis of variance (3-way ANOVA; groups, time, and leg; α < 0.05) followed by a two-way interaction analysis (α < 0.05). Analysis of the static and dynamic balance produced descriptive statistics, including the mean, standard deviation (SD), and standard error (SE).

## 3. Results

One hundred and two children started the program. Ninety-six of them attended all intervention sessions and were included in the study (94.1%).

The motor test results are presented in Table 1.

From the three-way ANOVA on the one-leg stance tests, no three-way interaction between groups, time (T0 vs. T2), or leg (right vs. left) was found (F(1,94) = 3.669, *p* = 0.058, η^2^ = 0.038). Moreover, no two-way interaction was found between leg and time (F(1,94) = 0.004, *p* > 0.05, η^2^ = 0.000) or between leg and group (F(1,94) = 0.4, *p* > 0.05, η^2^ = 0.004).

**Figure 1:**
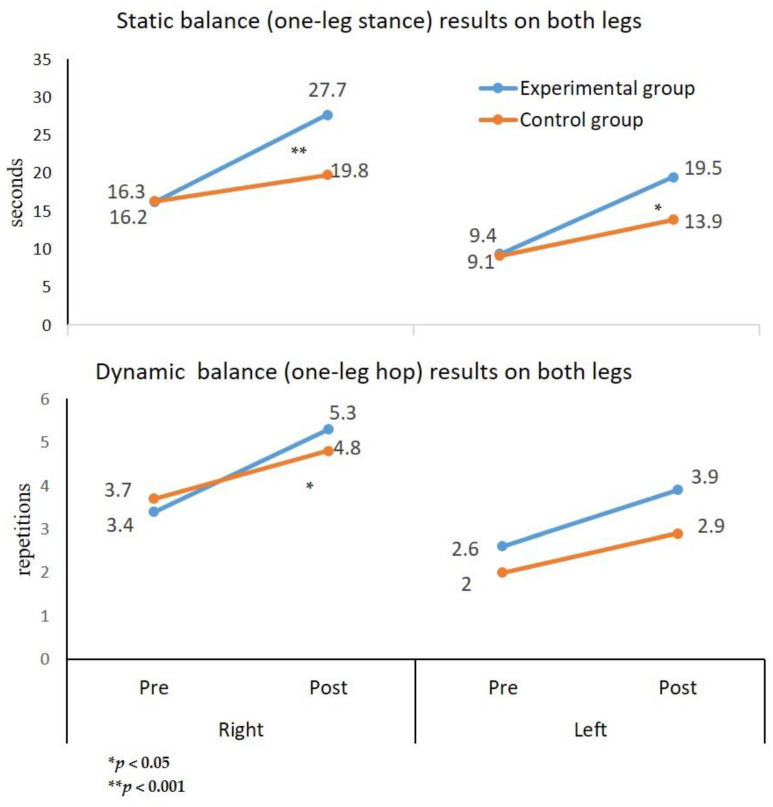
Results of time–group interaction of the static balance (one-leg stance in seconds) and dynamic balance (one-leg hop in repetitions) of both legs, pre- and post-intervention in both study groups (experimental vs. control).

An important finding is the time–group interaction (F(1,94) = 64.466, *p* < 0.001, η^2^ = 0.407), whereby the improvement in the one-leg stance in the experimental group following their dynamic and static balance training was greater than the improvement seen among the control group, which did not receive such training, as presented in Figure 1.

Additionally, participants in the experimental group significantly improved their one-leg stance on the right and left leg by 71% and 108%, respectively (*p* < 0.05), while participants in the control group tended to improve their one-leg stance on the right and left leg by only 22% and 53%, respectively.

Three-way ANOVA on the one-leg hop tests showed a tendency of an interaction between groups, time, and leg (F(1,94) = 1.291, *p* > 0.05, η^2^ = 0.014). However, a two-way interaction was seen between leg and time (F(1,94) = 4.113, *p* < 0.05, η^2^ = 0.042). Moreover, a significant time and group interaction (F(1,94) = 9.378, *p* < 0.05, η^2^ = 0.091) was evident, whereby the improvement in the one-leg hop test in the experimental group was greater than that of the control group (Figure 1). Additionally, participants in the experimental group improved their one-leg hop results on the right and left leg by 56% and 50%, respectively, while the participants in the control group improved their one-leg hop results on the right and left leg by 30% and 45%, respectively.

## 4. Discussion

A great amount of research has been conducted in the past few years in order to explore the impact of the COVID-19 pandemic on different aspects of life [17,19]. This study aimed to examine a means for improving the important skill of balance among young children who returned to kindergarten after a number of long lockdowns and periods of social isolation due to this emergency situation. The study hypothesized that specific balance training could contribute to improved static and dynamic balance performance among those children. The results of this study suggest that a short, designated treatment focused on balance has a beneficial influence on the static balance of preschoolers.

The COVID-19 pandemic crisis created a novel situation in which educational institutions were forced to close their doors, and children were required to stay indoors for long periods of time. As a result, deterioration was observed in various physical and cognitive aspects, with the sedentary pattern—which had already been documented prior to the crisis—increasing alarmingly [17,19]. Furthermore, children began to show significantly shorter single-leg standing time and a significantly greater number of falls per month following the onset of the emergency situation [20].

The short-term intervention program presented in this study was able to reverse some of the negative physical outcomes created by the long lockdowns. For example, the results of the one-leg stance showed an interaction between group and treatment, whereby the static stability of the experimental group improved significantly following the intervention training. This change cannot be explained by normal development alone, as the intervention only lasted five weeks, and such a change was not seen in the control group. As there is evidence that static balance tends to decrease between the end of kindergarten and the end of the first year at school (up to age 7) [22], these findings offer an easy and cost-effective solution. As such, after a long sedentary period, a short, specifically designed program may contribute to significant balance improvement.

Both static and dynamic balance were tested in this study. While the static balance of the experimental group improved significantly compared to that of the control group, an improvement was seen in the dynamic balance tests in both groups. It has been suggested that dynamic balance may improve through core stability training [26]. The control group in this study performed general physical activities that may have had a positive influence on some core muscles and, in turn, on the participants’ dynamic balance. More research may be needed to further address this point.

Balance skills are fundamental to all motor abilities, from the most basic movements to the most complex motor skills [5], and even constitute a connection point for certain cognitive processes, such as attention, concentration, and mental imagery [6]. The need to balance the body in stationary and mobile situations occurs frequently throughout the day. As such, it is important to train this skill from an early age. Intervention programs where balance plays a key role (e.g., yoga and “Minds in Motion”) were found to also contribute to the academic skills of elementary school children [27]. As such, when choosing a motor skill on which to focus, balance skills should be given preference.

The practical outcome of this study may be the possibility of designing short, cost-effective programs consisting of only a few sessions that can be used to restore deteriorated physical abilities. These programs may be used in times of lockdowns or other emergency situations. Although this study was focused on balance, further studies may target other motor abilities, such as coordination, kinesthesis, or even speed. Furthermore, future studies may be focused on restoring or improving balance or other abilities among children diagnosed with delay in their motor development, such as developmental coordination disorder (DCD).

Despite the importance of this research study and its outcomes, some limitations should be addressed. Although four kindergartens were included in the study, providing an adequate sample size, they were all part of the same educational institution. Moreover, this framework catered to families of strong financial and social standing. As such, future studies could benefit from comparing children from different institutions and from different backgrounds and statuses. In a time of uncertainty, such as during the COVID-19 pandemic, children tend to miss many days of school, especially when they or their family members test positive for the virus. As such, future studies should monitor the attendance of the children in the intervention program and offer an intervention program to those who are forced to stay remote.

## 5. Conclusions

In conclusion, this study showed that even a short-term targeted program can positively improve the motor abilities of preschoolers, especially after long periods of lockdown and social isolation. Moreover, such interventions can be applied with ease, even by kindergarten teachers who are not trained in physical education, without incurring additional resources such as time and money. Further research, however, is needed to fully understand the complete impact of the crisis on children’s balance and motor skills and how to best address this issue.

## Figures and Tables

**Table 1:** Motor test results pre- and post-intervention (mean ±SD).

Motor Test	Intervention	Experimental Group (N = 50)	Control Group (N = 46)
One-leg stance, right leg (seconds)	Pre	16.2 ± 8.6	16.3 ± 7.2
	* Post	*** 27.7 ± 9.9	19.8 ± 7.9
One-leg stance, left leg (seconds)	Pre	9.4 ± 5.1	9.1 ± 3.9
	* Post	*** 19.5 ± 5.6	13.9 ± 5.2
One-leg hop, right leg (repetitions)	Pre	3.4 ± 1.3	3.7 ± 1.1
	** Post	**** 5.3 ± 1.8	4.8 ± 1.5
One-leg hop, left leg (repetitions)	Pre	2.6 ± 1.5	2.0 ± 1.0
	** Post	3.9 ± 1.5	2.9 ± 1.3

* Significant time–group interaction. ** Significant time–leg interaction. *** Significant within group. **** Significant between groups.

## Data Availability

The data presented in this study are available on request from the corresponding author.

## Appendix A

Appendix A contains detailed information on the interventions and motor skills tests that were conducted in the study.

**Table A1:** Outline of the balance and control procedures.

S#	Group Focusing on Non-Balance Topics (control)	Group Focusing on Balance
**1**	**Meeting goals:** Learn concepts of space and spatial boundaries. **Sample tasks:** Playing the song "In our yard". Each time the word "peace" is heard in the song, the children will enter a hoop—the teacher explains that this is their personal space and asks what can be done in a personal space. The children stand "in their homes" (the hoops). When the music plays the children go out to skip in the general space (between the hoops), a space that belongs to everyone, and they learn behavioral rules in the common space.	**Meeting goals:** Improving static and dynamic balance using various bases and heights. **Sample tasks:** Move in space according to the music, stop in any way you like, and at any stop on other body parts. In an "all-four position" move on the hands and feet, change the directions of progress backwards, sideways. Raise another limb. Find other ways to balance your body on four support bases. Try to move on them. Try, in a small leg-spread, to detach the legs and turn a quarter turn, a half turn, and land without losing balance. Balance the body on one of the body parts (not on the legs!)– Buttocks, abdomen, back.
**2**	**Meeting goals:** The children will experience movement at different rhythms and at different height levels. **Sample tasks:** Progress in space at a slow pace at a low altitude level. Move forward at a rapid pace on the toes. Upon clapping move in a stooped position. Move forward in a creative way at a medium-height level	**Meeting goals:** Static balance with eyes closed and on an elevated platform. **Sample tasks:** Move between the stools. One knock—balance yourself on the stool. Two knocks—balance yourself on the floor. Stand on one leg on the stool and together we clap our hands up to the sky. Stand with one leg next to the stool, try to lift the stool and place it without lowering the other leg.
**3**	**Meeting goals:** The children will move their bodies in different ways and practice how to stop the movement. The children will move in space while considering their kindergarten counterparts. **Sample tasks:** Walking around the kindergarten. When the music stops, everyone stands like a statue, motionless. Walking backwards and standing next to a friend without contact when the music stops. Imitation dance—mimicking the teachers’ movements. Each time the song stops the children must stand still.	**Meeting goals:** Balancing the body on different fulcrums in different situations than usual. **Sample tasks:** Find a partner, try different swings. Sit back-to-back, combine elbows with your partner, bring your knees close to your chest, try to get up together. Lie on the floor and try to roll together like a "sausage" (straight body). Standing alone, try with a large legs-spread to balance on both hands and one knee. Stand and create a statue on the narrowest base while others (children or the teacher) try to unbalance you by pushing slightly.

Note. S#—session number.
